# Selected clinical parameters and changes in cardiac morphology and function assessed by magnetic resonance imaging in patients with rheumatoid arthritis and ankylosing spondylitis without clinically apparent heart disease

**DOI:** 10.1007/s10067-021-05777-6

**Published:** 2021-06-26

**Authors:** Wojciech Tański, Paweł Gać, Angelika Chachaj, Małgorzata Sobieszczańska, Rafał Poręba, Andrzej Szuba

**Affiliations:** 1 Department of Internal Medicine, 4th Military Hospital, Weigla 5, PL 50-981 Wroclaw, Poland; 2 Centre for Diagnostic Imaging, 4th Military Hospital, Weigla 5, PL 50-981 Wroclaw, Poland; 3 Department of Hygiene, Wroclaw Medical University, Mikulicza-Radeckiego 7, PL 50-368 Wrocław, Poland; 4 Department of Angiology, Hypertension and Diabetology, Wroclaw Medical University, Borowska 213, PL 50-556 Wrocław, Poland; 5 Department of Geriatrics, Wroclaw Medical University, Curie-Skłodowskiej 66, PL 50-369 Wrocław, Poland; 6 Department of Internal Medicine, Occupational Diseases and Hypertension, Wroclaw Medical University, Borowska 213, PL 50-556 Wrocław, Poland

**Keywords:** Ankylosing spondylitis, Cardiac magnetic resonance, Late gadolinium enhancement, Myocardium oedema, Neutrophil gelatinase–associated lipocalin, Rheumatoid arthritis

## Abstract

**Background:**

The aim of the study was to assess the relationship between the occurrence of rheumatoid arthritis (RA) and ankylosing spondylitis (AS) and the cardiac magnetic resonance (CMR) changes in people without clinically overt heart disease.

**Methods:**

The study group consisted of 74 people (48.81 ± 11.35 years): 29 patients with RA, 23 patients with AS and 22 people from control group. Blood samples were taken to assess laboratory parameters, disease activity was determined using activity scales, and CMR was performed.

**Results:**

It was shown that the factors independently related to higher left ventricular mass index are AS occurrence, human B27 leukocyte antigen occurrence, higher neutrophil gelatinase–associated lipocalin concentration (NGAL) and higher body mass index (BMI). The lower right ventricular ejection fraction is result of an independent effect of RA, AS and higher NGAL. RA presence, methotrexate use, higher rheumatoid factor titer, higher NGAL, older age and higher BMI should be considered independent risk factors for greater left ventricular myocardium water content. RA occurrence, AS occurrence, type 2 diabetes occurrence and a higher C-reactive protein concentration can be independently associated with a higher probability of non-ischemic left ventricular myocardium injury. Larger pericardial fluid volume is result of an independent effect of higher NGAL, higher anti-cyclic citrullinated peptide antibodies titer and higher DAS28 disease activity index. Use of steroids is protective factor against larger volume of pericardial fluid.

**Conclusions:**

RA and AS in people without clinically apparent heart disease are associated with the occurrence of adverse changes in CMR.

**Table:** 

**Key Points** •*RA and AS in people without clinically apparent heart disease are associated with the occurrence of adverse changes in CMR.*. •*The independent risk factors for higher LVEF are AS occurrence, human B27 leukocyte antigen occurrence, higher NGAL concentration and higher BMI.*. •*RA presence, methotrexate use, higher RF, higher NGAL, older age and higher BMI are independent risk factors for higher LV T2 ratio.*. •*RA occurrence, AS occurrence, type 2 diabetes occurrence and a higher CRP are independently associated with a higher risk of non-ischemic LV myocardium injury.*.

## Introduction

Cardiovascular diseases are the leading cause of death in the world [1]. The problem of the prevalence of cardiovascular diseases is also more applicable to areas with high gross domestic product [2, 3]. In addition to primary cardiovascular diseases, cardiac and vascular pathological changes may be secondary to diseases of other organs and organ systems [4, 5].

Rheumatoid arthritis (RA) and ankylosing spondylitis (AS) are one of the most common rheumatic diseases [6, 7]. Coronary artery disease, subclinical and apparent cardiovascular disease, pericarditis, myocarditis, valvular disease, vasculitis and secondary cardiomyopathy are more common in patients with RA [8–10]. Data regarding AS indicate similar relationships, but they are less documented and less expressed [11].

Cardiac magnetic resonance (CMR) is currently the “golden standard” of assessing left and right ventricular function and enables non-invasive assessment of myocardial oedema and fibrosis [12–14].

The previously identified relationships between RA, AS, and cardiovascular disease were mainly observed in observational epidemiological studies [15, 16]. However, among the imaging methods, cardiovascular changes in patients with RA and AS have so far been assessed primarily using echocardiography [17].

The aim of the study was to assess the relationship between the occurrence of RA and AS and the morphology and function of the heart assessed by CMR in people without clinically overt heart disease. In addition, an attempt was made to link selected clinical parameters of RA and AS to specific morphological and functional changes of the heart with CMR.

## Materials and methods

The entire study group consisted of 74 people (48.81 ± 11.35 years). The study protocol included the inclusion of consecutive patients with diagnosed RA or AS, studied in the department of internal medicine in order to assess organ complications, treatment effectiveness, and possible optimization therapy. Patients with clinically apparent heart diseases were not qualified for the study. Patients who were enrolled in the project did not report any clinical symptoms that would indicate heart disease; they had not been diagnosed with heart disease in previous studies, including additional studies. In the first stage, 29 patients with RA and 23 patients with AS were qualified for the study. In the next stage, the study group included people not suffering from rheumatic diseases constituting the control group. Finally, 22 people were qualified to the control group. The general characteristics of the studied groups are given in Table 1. Selected clinical parameters in the groups with RA and AS are summarized in Tables 2 and 3.

**Table 1:** The general characteristics of the studied groups

	RA	AS	Control
Number [n/%]	29 / 100.0	23 / 100.0	22 / 100.0
Gender [n/%]			
Men	9 / 31.0***	23 / 65.2	10 / 45.4
Women	20 / 69.0***	8 / 34.8	12 / 54.6
Age [years]	49.41 ± 13.72	47.11 ± 11.29	49.40 ± 7.73
Height [m]	1.65 ± 0.08	1.64 ± 0.09	1.69 ± 0.07
Weight [kg]	71.20 ± 13.80	67.50 ± 12.85	71.82 ± 9.60
Body mass index (BMI) [kg/m^2^]	25.68 ± 4.02	25.10 ± 3.93	25.19 ± 3.05
Body surface area (BSA) [m^2^]	1.76 ± 0.17	1.73 ± 0.18	1.82 ± 0.13
Overweight/obesity [n/%]			
Normal body mass	13 / 44.8	9 / 39.1	12 / 54.6
Overweight	9 / 31.0	11 / 47.8	8 / 36.3
Obesity	7 / 24.2	3 / 13.0	2 / 9.2
Coexistence of cardiovascular risk factors [n/%]			
Arterial hypertension	7 / 24.2	2 / 8.6	3 / 18.7
Type 2 diabetes	3 / 10.3	1 / 4.3	1 / 4.5
Smoking	2 / 6.9	1 / 4.3	3 / 13.6

^***^ RA vs. AS: *p* < 0.05

**Table 2:** Selected clinical parameters in patients with rheumatoid arthritis

Duration of illness [years]	8.40 ± 6.67
C-reactive protein (CRP) [mg/l]	17.96 ± 17.32
Rheumatoid factor (RF) [IU/ml]	170.74 ± 336.04
Anti-cyclic citrullinated peptide (anti-CCP) [EU/ml]	326.46 ± 488.88
Seropositivity [n/%]	19 / 65.5
Disease activity index DAS28	6.49 ± 0.53
Collective assessment of health by the patient and the doctor (VAS)	82.67 ± 4.17
Neutrophil gelatinase–associated lipocalin (NGAL) [ng/ml]	13.22 ± 8.77
Steroids [n/%]	19 / 65.5
Methotrexate [n/%]	21 / 72.4
Other disease-modifying drugs [n/%]	11 / 37.9
Salazopyrin	1 / 3.4
Leflunomide	6 / 20.7
Cyclosporine	3 / 10.3
Chloroquine	1 / 3.4

**Table 3:** Selected clinical parameters in patients with patients with ankylosing spondylitis

Duration of illness [years]	9.48 ± 10.49
C-reactive protein [mg/l]	24.04 ± 28.26
B27 human leukocyte antigen (HLA-B27) [n/%]	18 / 78.26
Neutrophil gelatinase–associated lipocalin (NGAL) [ng/ml]	23.15 ± 22.55
Disease-modifying drugs [n/%]	16 / 69.5
Etanercept	6 / 26.1
Adalimumab	7 / 30.4
Secukinumab	3 / 13.0

Based on the history, basic information on the underlying disease and comorbidities was collected, including data on the prevalence of risk factors for cardiovascular disease. Pharmacological preparations used at the time of the study were also identified.

In venous blood in the group with RA, C-reactive protein (CRP) concentrations, rheumatoid factor (RF) titers, anti-cyclic citrullinated peptide (anti-CCP) titers and neutrophil gelatinase–associated lipocalin (NGAL) concentration were determined; seropositivity was also determined. In the group with AS, the levels of CRP and the concentration of NGAL were determined; in addition, a B27 human leukocyte antigen (HLA-B27) test was performed. Laboratory analyses were performed using commercially available tests in a laboratory diagnostics facility controlled as part of the Randox International Quality Assessment Scheme (RIQAS).

In patients with RA, disease activity was assessed using two standardized methods: disease activity index DAS28 and VAS collective assessment of health by the patient and the doctor.

CMR tests were performed with one 1.5 T Magnetom Aera (Siemens Healthcare, Forchheim, Germany). All CMR tests were performed according to a standardized protocol. The image acquisition was ECG-gated with breath holds. The study protocol included CINE-type steady-state free precession sequences, short-tau inversion-recovery sequences (STIR) and late gadolinium enhancement sequences (LGE). The LGE sequence was performed after injection through the veins of the ulnar bolus gadobutrol dose 0.2 mmol/kg body weight (Gadovist, Bayer Healthcare, Leverkusen, Germany). Medis Suite MR software (Medis, Leiden, the Netherlands) was used for post-processing evaluation of CMR images.

CINE sequences were recorded in the short axis of the left ventricle and in the long axis in 2-chamber, 3-chamber and 4-chamber projections. CINE sequences were used to assess the dimensions of the heart cavities and the functional parameters of the left and right ventricles. The 4-chamber projection assessed the left atrial surface area (LAA) and the right atrial surface area (RAA). In the short axis projection in the baso-median slices, left ventricular end-diastolic diameter (LVEDD), left ventricular end-systolic diameter (LVESD), anterior intra-ventricular septum diastolic diameter (aIVSDD) and posterior wall diastolic diameter (PWDD) were measured. The left ventricular fractional shortening (LV FS) was calculated using the formula: FS = (LVEDD – LVESD / LVEDD) *100%. 2-chamber projections in the long axis and images in the short axis of the left ventricle were used to estimate the parameters of left and right ventricular function using the volumetric method. The end-diastolic and end-systolic volume (EDV and ESV) was calculated as the sum of the left ventricular cavity surface in subsequent layers in images in the short axis multiplied by the thickness of the layer. Stroke volume (SV) was the difference between EDV and ESV. The ejection fraction (EF) was obtained by stroke volume divided by end-diastolic volume. Functional parameters of the left and right ventricles (LV and RV) are presented as body surface area (BSA) indexed values. Left ventricular mass index (LVMI) was calculated too.

STIR and LGE sequences were performed to assess the morphological changes of myocardium. The occurrence of left and right ventricular oedema foci was assessed, and the number of left ventricular segments with visualized oedema foci and the planimetrically measured surface area of the largest oedema focus were determined. In addition, the T2 ratio was measured, i.e. the ratio of maximum myocardial intensity to skeletal muscle intensity. In the absence of visible oedema foci, the T2 ratio was used to determine the occurrence of generalized oedema of the left ventricular myocardium. The normal T2 ratio was < 1.8, and the criterion for generalized oedema of the left ventricular myocardium was T2 ratio > 1.9. Values between 1.8 and 1.9 were considered borderline for generalized left ventricular myocardial oedema.

LGE foci of left and right ventricle were identified; the number of left ventricular segments with visualized LGE foci and the surface area of the largest LGE focus was measured by planimetry. In the case of LGE foci, the ratio of the intensity of the largest LGE focus to the intensity of myocardium T1 ratio was calculated. In the next stage, LGE foci were classified in terms of location as transmural, sub-epicardial, intramural and subendocardial. The presence of intramural and/or subendocardial LGE foci made it possible to recognize ischemic injury to the left ventricular myocardium, while the occurrence of sub-epicardial and intramural LGE foci — non-ischemic injury to myocardium.

In addition, the presence of pericardial fluid was included in the CMR assessment. In the presence of pericardial fluid, maximum pericardial fluid was measured.

Statistical calculations were made using the “Dell Statistica 13” statistical software (Dell Inc., USA). Quantitative variables are presented as arithmetic means ± standard deviations. The distribution of variables was checked using the W-Shapiro–Wilk test. For independent quantitative variables with normal distribution, ANOVA one-way parametric analysis of variance was used. For variables with a non-normal distribution, the non-parametric equivalent of variance analysis ANOVA Kruskal–Wallis test was used. The results for qualitative variables are presented as percentages. For independent qualitative variables, the chi-square test of maximum likelihood was used for further analysis. To assess the relationship between the examined parameters, a correlation and regression analysis was performed. Sensitivity and specificity analysis were performed using ROC curve analysis. The results at the level of *p* < 0.05 were considered statistically significant.

## Results

Parameters of cardiac magnetic resonance in the studied groups of patients are presented in Table 4. In terms of heart cavity dimensions, the group with RA was characterized by significantly higher PWDD compared to the control group (8.50 ± 1.53 mm vs. 7.18 ± 1.23 mm), while the group with AS has a significantly higher LVMI than the control group (68.40 ± 24.94 g/m^2^ vs. 61.12 ± 15.26 g/m^2^). Both examined groups of patients in relation to the control group had worse right ventricular systolic function expressed in significantly lower RV EF (RA: 55.80 ± 2.05%, AS: 55.31 ± 1.81%, CON: 62.15 ± 7.00%).

**Table 4:** Parameters of cardiac magnetic resonance in the studied groups of patients

	RA	AS	Control
Heart cavities dimensions			
Left atrium surface area in 4-chamber projection (LAA) [cm^2^]	23.32 ± 5.14	24.75 ± 3.60	23.84 ± 4.04
Right atrium surface area in 4-chamber projection (RAA) [cm^2^]	19.81 ± 3.90	20.77 ± 4.06	22.23 ± 3.21
Left ventricular end-diastolic diameter (LVEDD) [mm]	56.21 ± 31.28	56.91 ± 6.92	58.11 ± 8.30
Left ventricular end-systolic diameter (LVESD) [mm]	32.86 ± 12.10	34.09 ± 6.79	34.82 ± 10.48
Left ventricular fractional shortening (LV FS) [%]	38.79 ± 12.91	35.61 ± 7.45	36.01 ± 8.56
Anterior intra-ventricular septum diastolic diameter (aIVSDD) [mm]	9.18 ± 1.87	9.30 ± 2.01	8.44 ± 1.53
Posterior wall diastolic diameter (PWDD) [mm]	8.50 ± 1.53*	7.91 ± 1.70	7.18 ± 1.23
Left ventricular mass index (LVMI) [g/m^2^]	60.13 ± 12.10	68.40 ± 24.94**	61.12 ± 15.26
Left ventricular systolic function			
End-diastolic volume index (EDVi) [ml/m^2^]	81.90 ± 19.51	86.36 ± 19.35	85.08 ± 14.60
End-systolic volume index (ESVi) [ml/m^2^]	32.00 ± 12.41	27.55 ± 9.65	34.48 ± 12.48
Stroke volume index (SVi) [ml/m^2^]	49.90 ± 10.63	53.95 ± 12.44	56.83 ± 13.96
Ejection fraction (EF) [%]	65.25 ± 7.82	66.70 ± 4.72	63.06 ± 5.41
Reduced ejection fraction [n/%]	2 / 7.4	0 / 0.0	0 / 0.0
Right ventricular systolic function			
End-diastolic volume index (EDVi) [ml/m^2^]	98.85 ± 26.28	91.50 ± 27.35	93.44 ± 19.24
End-systolic volume index (ESVi) [ml/m^2^]	43.62 ± 11.61	40.85 ± 12.09	35.08 ± 9.15
Stroke volume index (SVi) [ml/m^2^]	55.22 ± 14.94	50.65 ± 15.40	58.36 ± 14.31
Ejection fraction (EF) [%]	55.80 ± 2.05*	55.31 ± 1.81**	62.15 ± 7.00
Reduced ejection fraction [n/%]	0 / 0.0	0 / 0.0	0 / 0.0
Myocardium oedema			
Left ventricular oedema foci [n/%]	1 / 3.4	0 / 0.0	0 / 0.0
Number of left ventricular segments with oedema foci	0.17 ± 0.93	0.00 ± 0.00	0.00 ± 0.00
Surface area of the largest oedema focus [cm^2^]	2.98 ± 0.00	-	-
The ratio of myocardial intensity to skeletal muscle intensity (T2 ratio)	1.71 ± 0.44*	1.61 ± 0.24	1.49 ± 0.17
Normal T2 ratio [n/%]	24 / 82.7*	20 / 87.0	100 / 100.0
Borderline image for generalized left ventricular myocardial oedema [n/%]	2 / 6.9	1 / 4.3	0 / 0.0
Generalized left ventricular myocardial oedema [n/%]	3 / 10.3	2 / 8.7	0 / 0.0
Right ventricular oedema foci [n/%]	1 / 3.4	0 / 0.0	0 / 0.0
Late gadolinium enhancement foci (LGE)			
Left ventricular LGE foci [n/%]	5 / 17.2*	3 / 13.0**	0 / 0.0
Number of left ventricular segments with LGE foci	0.86 ± 2.07*	0.48 ± 1.34	0.00 ± 0.00
Number of left ventricular segments with transmural LGE foci	0.00 ± 0.00	0.00 ± 0.00	0.00 ± 0.00
Number of left ventricular segments with subendocardial LGE foci	0.07 ± 0.37	0.00 ± 0.00	0.00 ± 0.00
Number of left ventricular segments with subepicardial LGE foci	0.17 ± 0.60	0.17 ± 0.65	0.00 ± 0.00
Number of left ventricular segments with intramural LGE foci	0.72 ± 1.67*	0.39 ± 1.08	0.00 ± 0.00
Surface area of the largest LGE focus [cm^2^]	0.76 ± 0.31	0.45 ± 0.08	-
Tthe ratio of the intensity of the largest LGE focus to the intensity of myocardium (T1 ratio)	3.49 ± 0.58	2.68 ± 0.98	-
Right ventricular LGE foci [n/%]	1 / 3.4	0 / 0.0	0 / 0.0
Ischemic myocardium injury [n/%]	1 / 3.4	0 / 0.0	0 / 0.0
Non-ischemic myocardium injury [n/%]	5 / 17.2*	3 / 13.0**	0 / 0.0
Pericardial fluid			
Presence of pericardial fluid [n/%]	7 / 24.1	3 / 13.0	2 / 9.1
Maximum pericardial plaque separation [mm]	7.50 ± 1.87*	9.33 ± 2.52**	2.50 ± 0.71

^*^ RA vs. Control: *p* < 0.05; ** AS vs. Control: *p* < 0.05

In the assessment of myocardial oedema, in the group with RA, the T2 ratio was statistically significantly higher than in the control group (1.71 ± 0.44 vs. 1.49 ± 0.17). At the same time, the percentage of patients with normal T2 ratio was significantly lower in the group with RA compared to the control group (82.7% vs. 100.0%). In the assessment of LGE, there was statistically significantly more frequent occurrence of post-contrast LGE enhancement in the groups with RA and AS than in the control group (RA: 17.2%, AS: 13.0%, CON: 0.0%). The nature of LGE foci in the examined groups allowed us to show a significantly more frequent occurrence of non-ischemic injury to myocardium in the groups with RA and AS in comparison with the control group (RA: 17.2%, AS: 13.0%, CON: 0.0%). RA patients were also characterized by a higher degree of myocardial fibrosis (expressed in the number of myocardial segments with LGE foci) than the control group (0.86 ± 2.07 vs. 0.00 ± 0.00). In RA patients a significantly larger number of myocardium segments with visualized intramural LGE foci than those in the control group (0.72 ± 1.67 vs. 0.00 ± 0.00).

In the presence of fluid in the pericardial sac, the maximum separation of pericardial plaques was statistically significantly higher in the groups with AS and RA compared to the control group (RA: 7.50 ± 1.87 mm, AS: 9.33 ± 2.52 mm, CON: 2.50 ± 0.71 mm).

In the correlation analysis, among the patients with RA, the strongest statistically linear dependency was found between: RF and indexed RV ESV (r = 0.70, *p* < 0.05), RF and indexed RV EDV (r = 0.64, *p* < 0.05) and anti-CCP and aIVSDD (r = 0.60, *p* < 0.05). In patients with AS, the highest values of correlation coefficients concerned the linear dependency between NGAL and the number of left ventricular segments with visualized LGE sub-epidural foci (r = 0.78, *p* < 0.05), NGAL and the number of left ventricular segments visualized by any LGE foci (r = 0.73, *p* < 0.05) and also between CRP and LV FS (r =  − 0.73, *p* < 0.05).

With the help of multivariable backward stepwise regression analysis, independent factors associated with the occurrence of recognized adverse CMR changes were estimated, i.e. greater LVMI, smaller left and right ventricular EF, greater T2 ratio of myocardial intensity to skeletal muscle intensity and greater maximum separation of pericardial plaques. In the logistic regression analysis, however, independent factors associated with non-ischemic injury to myocardium were identified. The parameters of the developed regression models are given in Table 5. Based on the obtained models, it was shown that in the examined group, the factors independently related to the higher LVMI are occurrence of AS, occurrence of human B27 leukocyte antigen, higher NGAL and higher BMI (Table 5). The lower RV EF in the study group is independently associated with RA, AS and higher NGAL (Table 6). In this group of patients, the presence of RA, current methotrexate use in therapy, higher RF, higher NGAL, older age and higher BMI should be considered independent risk factors for greater left ventricular myocardium water content (Table 7). In the same group, the occurrence of RA, the presence of AS, the occurrence of type 2 diabetes and a higher CRP can be independently associated with a higher probability of non-ischemic injury to left ventricular myocardium (Table 8). However, the larger volume of fluid in the pericardial sac in the subjects is the effect of the independent effect of higher NGAL, higher anti-CCP and higher DAS28. Current use of steroids in therapy is a protective factor against a larger volume of pericardial fluid assessed in CMR in the examined group (Table 9).

**Table 5:** Results of regression analysis in the studied group of patients. Risk factors for higher left ventricular mass index (LVMI)

Model for left ventricular mass index (LVMI) [g/m^2^]					
	Intercept	B27 human leukocyte antigen (HLA-B27)^#^	Body mass index (BMI) [kg/m^2^]	Ankylosing spondylitis^#^	Neutrophil gelatinase–associated lipocalin (NGAL) [ng/ml]
Regression coefficient	48.548	48.548	48.548	48.548	48.548
SEM of Rc	37.735	37.735	37.735	37.735	37.735
p	1.866	1.866	1.866	1.866	1.866

^#^ dichotomic variable, where 1- missing, 2- occurrence

**Table 6:** Results of regression analysis in the studied group of patients. Risk factors for lower right ventricular ejection fraction (RVEF)

Model for right ventricular ejection fraction (RVEF) [%]				
	Intercept	Rheumatoid arthritis^#^	Ankylosing spondylitis^#^	Neutrophil gelatinase–associated lipocalin (NGAL) [ng/ml]
regression coefficient	67.922	− 4.321	− 4.086	− 0.074
SEM of Rc	20.170	1.069	1.130	0.051
p	< 0.05	< 0.001	< 0.001	< 0.05

^#^ dichotomic variable, where 1- missing, 2- occurrence

**Table 7:** Results of regression analysis in the studied group of patients. Risk factors for higher left ventricular myocardium water content (T2 ratio)

Model for: the ratio of myocardial intensity to skeletal muscle intensity (T2 ratio)							
	Intercept	Rheumatoid factor (RF) [IU/ml]	Rheumatoid arthritis^#^	Neutrophil gelatinase–associated lipocalin (NGAL) [ng/ml]	Body mass index (BMI) [kg/m^2^]	Age [years]	Methotrexate^#^
Regression coefficient	− 0.087	0.001	0.373	0.008	0.021	0.005	0.174
SEM of Rc	0.174	0.000	0.095	0.003	0.009	0.002	0.089
p	< 0.05	< 0.001	< 0.001	< 0.001	< 0.001	< 0.001	< 0.001

^#^ dichotomic variable, where 1- missing, 2- occurrence

**Table 8:** Results of regression analysis in the studied group of patients. Risk factors for non-ischemic left ventricular myocardium injury

Model for: probability non-ischemic myocardium injury				
	Rheumatoid arthritis^#^	Ankylosing spondylitis^#^	Type 2 diabetes^#^	C-reactive protein (CRP) [mg/l]
Regression coefficient	13.103	10.482	1.143	5.017
SEM of Rc	2.156	2.145	0.248	1.023
p	< 0.01	< 0.01	< 0.01	< 0.01

^#^ dichotomic variable, where 1- missing, 2- occurrence

**Table 9:** Results of regression analysis in the studied group of patients. Risk factors for higher volume of pericardial fluid

Model for: maximum pericardial plaque separation [mm]					
	Intercept	Neutrophil gelatinase–associated lipocalin (NGAL) [ng/ml]	Anti-cyclic citrullinated peptide (anti-CCP) [EU/ml]	Steroids^#^	Disease activity index DAS28
Regression coefficient	7.005	0.155	0.004	- 2.156	3.759
SEM of Rc	1.245	0.032	0.001	0.697	1.289
p	< 0.01	< 0.001	< 0.01	< 0.01	< 0.01

^#^ dichotomic variable, where 1- missing, 2- occurrence

Using the ROC curve analysis and association analysis, the sensitivity and specificity of various clinical indicators were assessed as predictive indicators of the occurrence of generalized left ventricular myocardial oedema, non-ischemic injury to left ventricular myocardium and pericardial fluid in RA and AS. The following criteria can be considered tests with high sensitivity and specificity of prediction of generalized left ventricular myocardial oedema in RA: NGAL > 19.6 ng/ml and RA duration > 10 years. The following criteria are corresponding tests for non-ischemic left ventricular myocardial injury in RA patients: NGAL > 15.5 ng/ml and anti-CCP > 393.6 EU/ml. In the case of prediction of pericardial fluid, the following criteria should be considered: anti-CCP > 284.6 EU/ml and CRP > 18.8 mg/l. The results of the analysis of the sensitivity and specificity of selected clinical parameters as predictors of CMR in the group with RA are presented in Table 10.

**Table 10:** Sensitivity and specificity of selected clinical parameters as predictors of cardiac magnetic resonance changes in patients with rheumatoid arthritis

		Duration of illness>10 years^*^	CRP>28.9 mg/l	RF>33.7 IU/ml	Anti-CCP>393.6 EU/ml	DAS28 >6.3	VAS>85	NGAL>19.6ng/ml^*^
Prediction of generalized left ventricular myocardial oedema	Sensitivity	0.731	0.731	0.421	0.667	0.133	0.846	0.833
	Specificity	0.667	0.000	0.667	0.333	1.000	0.500	0.667
	Accuracy	0.724	0.655	0.455	0.625	0.235	0.800	0.810
		Duration of illness>3 years	CRP>23.0 mg/l	RF>1480.0 IU/ml	Anti-CCP>393.6 EU/ml^*^	DAS28>7.5	VAS>85	NGAL >15.5 ng/ml^*^
Prediction of non-ischemic myocardial injury	Sensitivity	0.391	0.696	0.947	0.737	1.000	0.833	0.733
	Specificity	0.833	0.333	0.000	0.600	0.333	0.333	0.667
	Accuracy	0.483	0.621	0.818	0.708	0.882	0.733	0.714
		Duration of illness > 7 years	CRP > 18.8 mg/l^*^	RF > 9.75 IU/ml	Anti-CCP >284.6 EU/ml^*^	DAS28 >7.5	VAS > 90	NGAL >13.0 ng/ml
Prediction of pericardial fluid	Sensitivity	0.500	0.682	0.353	0.647	1.000	0.800	0.600
	Specificity	0.429	0.571	0.364	0.833	0.167	0.200	0.500
	Accuracy	0.483	0.655	0.400	0.696	0.706	0.600	0.571

^*^ statistically useful prediction test

In the group with AS, the criteria based on the assessed clinical indicators are not characterized by sufficiently high sensitivity and specificity prediction of generalized myocardial oedema and non-ischemic left ventricular myocardial injury. On the other hand, the following criteria can be considered tests with high sensitivity and specificity of the occurrence of pericardial fluid in AS: AS duration > 4 years and CRP > 12.1 mg/l. Full results of the analysis of the sensitivity and specificity of selected clinical parameters as predictors of CMR in the group with AS are presented in Table 11.

**Table 11:** Sensitivity and specificity of selected clinical parameters as predictors of cardiac magnetic resonance changes in patients with ankylosing spondylitis

		Duration of illness>31 years	CRP>20.3 mg/l	NGAL >21.5 ng/ml
Prediction of generalized left ventricular myocardial oedema	Sensitivity	0.905	0.684	1.000
	Specificity	0.000	0.000	0.500
	Accuracy	0.826	0.619	0.875
		Duration of illness> 4 years	CRP>17.7 mg/l	NGAL>21.5ng/ml
Prediction of non-ischemic myocardial injury	Sensitivity	0.550	0.667	1.000
	Specificity	0.333	0.333	0.333
	Accuracy	0.522	0.619	0.750
		Duration of illness> 4 years^*^	CRP>12.1 mg/l^*^	NGAL>16.3 ng/ml
Prediction of pericardial fluid	Sensitivity	0.600	0.556	0.600
	Specificity	0.667	0.667	0.333
	Accuracy	0.609	0.571	0.500

^*^ statistically useful prediction test

## Discussion

Based on the performed study, it was shown that in study group, RA and AS are associated with the occurrence of adverse changes in morphology and cardiac function assessed by CMR, such as worse right ventricular systolic function, a greater degree of myocardial oedema as measured by T2, greater severity of non-ischemic myocardial fibrosis and larger pericardial effusion. The existence of certain relationships between RA and AS and the dimensions of the heart cavities have also been documented. In addition, the results of regression analysis are also confirmed by the existence of the above relationships between RA and AS and morphological and functional changes of the heart in CMR in study group.

The more frequent occurrence and greater severity of adverse cardiovascular changes in patients with RA in our study compared to the control group matches the results of other studies conducted so far, although most of them were conducted using echocardiography [18]. There are far fewer CMR studies. In the research of Ntusi et al., compared to the control group, RA patients were characterized by significantly larger left atrium sizes, worse left ventricular contractility rates, larger myocardial oedema foci, more frequent myocardial fibrosis foci, higher myocardial T1 native time, greater left ventricular myocardium volume percentage with time T1 > 990 ms in T1-mapping images and a larger volume of extracellular space [19]. In the study of Kobayashi et al. of 2010, the frequency of CMR changes in RA patients was estimated at 45% of cases. LGE foci corresponding to areas of myocardial fibrosis were identified in 39% of patients with RA. In 11% of patients, myocardial perfusion defects in left ventricle were observed [20]. Later studies by the same team of authors performed on a larger group of RA patients indicated that left ventricular fibrosis foci occurs in 32% of patients; in 12% of cases myocardial inflammatory activity can be shown in T2-dependent imaging [21]. The results of current study seem to be consistent with the discussed studies of other authors. Interesting results were provided by a study by Giles et al., in which RA was associated with a smaller left ventricular EF and a smaller LVMI [22]. The results of our study did not confirm the existence of such a dependency. Both LV EF and LVMI did not differentiate the control group from the group of patients with RA.

Demonstration of the dependency between AS and the occurrence and greater severity of adverse cardiovascular changes in this study is a valuable complement to existing studies. In the study of Biesbroek et al. conducted in the group of 15 patients, the incidence of left ventricular systolic dysfunction was estimated to be 36%, the incidence of focal left ventricular myocardial fibrosis was 21%, and the volume of extracellular myocardial space was linked to disease activity [23]. In other studies, by the same researchers, in patients with AS, using CMR adverse changes in cardiac morphology and function were linked to increased aortic arch stiffness [24]. Our study matches the realization of the postulate of the need to disseminate awareness of the possibility of reducing the incidence of cardiovascular events and improving the survival of patients with AS when early diagnosis of cardiovascular changes [25].

The currently discussed study may also indicate that in patients without clinically apparent myocardial injury during RA and AS, there occur corresponding CMR-assessed changes in heart morphology and function. As already mentioned, previous studies on the relation between RA and AS and adverse morphological and functional changes in the heart showed similarities in the demonstrated dependencies, however, in the case of AS, the dependencies were less well documented and, above all, were to be less expressed [17]. According to the authors, the results of our study are a valuable supplement to the data in the context of comparing patients with RA and AS.

The analysis of the results of the currently discussed study also allows to conclude on the usefulness of selected clinical and laboratory parameters characterizing the severity and course of RA and AS for the purpose of predicting the risk of adverse changes in CMR. As it has been shown that there are some linear relationships between clinical parameters disease and selected CMR parameters. Predictive usefulness of RA and AS clinical and laboratory parameters included in the study for morphological and functional changes of the heart in CMR was confirmed by regression analysis. Based on the above data, NGAL concentration should be considered the most useful clinical parameter for the purpose of predicting the risk of adverse changes in CMR in the studied group of patients with RA and AS.

NGAL is primarily an early marker of acute kidney injury. In recent years, however, it has been shown that it can be an indicator of increased cardiovascular risk [26]. The importance of NGAL in rheumatic diseases was looks into a few years ago [27]. Studies on the relationship between NGAL and CMR changes have not been conducted so far, and the relationships shown in the current study should be considered as an important premise for more extensive research in this area.

Out of the clinical parameters assessed in the present study, in previous scientific studies the issue of the relationship between them and changes found in CMR was raised in relation to the duration of the disease, anti-CCP, CRP and DAS28. In the study of Bradham et al., no relationship between disease duration, CRP concentration and DAS28 index and native T1 time and extracellular volume of the left ventricular myocardium in RA [28] was indicated. The predictive significance of CRP in RA was raised in the studies of Kobayashi et al. An increase in the CRP by a log transformed was associated with a 3.36-fold increase in the risk of LGE foci in the left ventricular myocardium [21]. In earlier studies, in the context of adverse CMR changes, the importance of CRP and DAS28 index was indicated, at the same time in the absence of such significance for the duration of the disease and the status of autoantibodies. Patients with diagnosed LGE foci in the left ventricular myocardium were characterized by higher CRP and DAS28 index [29]. Still other studies, namely those carried out by Giles et al., emphasized the predictive significance of anti-CCP, in the absence of other indicators of RA activity and severity. Anti-CCP was associated with significantly lower LVM, LV EDV and LV SV, but not with LV EF [22]. The dependencies shown in our research may contribute to a more complete understanding of the above dependencies.

The discussed studies allow to infer about the dependency between the current RA treatment and the occurrence of CMR changes. Using regression analysis, it was shown that the methotrexate use is an independent factor associated with greater water content of the left ventricle myocardium, and the steroid use is an independent factor associated with smaller pericardial plaque separation by pericardial fluid.

In previous studies, the issue of adverse cardiological consequences of using methotrexate therapy has already been signaled, even in studies by Kobayashi et al. [29]. The authors postulated the need for further research on the potential cardiotoxicity of methotrexate, noting the existence of contrasting reports on the relationship between the use of methotrexate and the reduction of cardiovascular risk [29, 30]. In the context of the results of our study, this postulate seems most justified.

The dependency between the use of steroids for inflammatory diseases and the smaller volume of fluid when it occurs in the pericardial sac appears to be consistent with the literature. It was reflected, among others, in the current guidelines of the European Society of Cardiology on the diagnosis and treatment of pericardial diseases of 2015, in the algorithm of treatment in the presence of pericardial fluid [31].

A somewhat surprising result of our study is the demonstration of a statistically significantly lower right ventricular ejection fraction in patients with RA and AS than in the control group, with no significant differences in the left ventricular ejection fraction. In previous studies, both left and right ventricular systolic dysfunction have been demonstrated in patients with RA and AS, but with a predisposition to the earlier occurrence of left ventricular systolic dysfunction [32–34]. It should be noted that many of such examinations were conducted using the echocardiographic method, and our study used the “gold standard” for the assessment of left and right ventricular function, which is magnetic resonance imaging. Moreover, it should be clearly emphasized that in our study, despite a statistically significant lower value of the right ventricular ejection fraction in patients with RA and AS than in the control group, its absolute value in these groups was within the normative values.

The study is obviously not without significant limitations. The basic methodological limitation of the study is the lack of echocardiographic examination in patients qualified for the project. The small size of the studied groups should also be considered a significant limitation. It would be optimal in qualifying patients for the study not only to omit patients with clinically apparent myocardial injury, but also to omit patients with risk factors for cardiovascular disease. Questions may arise regarding the selection of laboratory parameters in RA and AS. The CMR could be supplemented with mapping sequences.

## Conclusions

1. RA and AS in people without clinically apparent myocardial injury are associated with the occurrence of adverse changes in morphology and cardiac function assessed by CMR, primarily, worse right ventricular systolic function, a greater degree of myocardial water content, greater severity of non-ischemic myocardial fibrosis and pericardial effusion.

2. In patients without clinically apparent myocardial injury, during RA and AS, there occur similar changes in heart morphology and function assessed using CMR, with similar severity.

3. NGAL concentration is the most useful clinical parameter among the subjects for the purpose of predicting the risk of adverse CMR changes in the studied group of patients with RA and AS.

## Additional information

The research results were presented at the European Congress of Radiology: ECR 2020. [Digital only. July 15–19, 2020]. The abstract was published in the conference materials: Insights Imaging (2020) 11 (Suppl 1): 34, p. 286, item RPS 603b-9. https://link.springer.com/content/pdf/10.1186/s13244-020-00851-0.pdf

## References

[1] WHO (2016). Global health estimates 2015: Deaths by cause, age, sex, by country and by region, 2000–2015.

[2] Roth GA, Forouzanfar MH, Moran AE, Barber R, Nguyen G, Feigin VL, Naghavi M, Mensah GA, Murray CJ (2015). Demographic and epidemiologic drivers of global cardiovascular mortality. N Engl J Med.

[3] Al-Kindi S, Al-Juhaishi T, Haddad F, Taheri S, Abi Khalil C (2015). Cardiovascular disease research activity in the Middle East: a bibliometric analysis. Ther Adv Cardiovasc Dis.

[4] Generali E, Folci M, Selmi C, Riboldi P (2017). Immune-mediated heart disease. Adv Exp Med Biol.

[5] Eriksson U, Penninger JM (2005). Autoimmune heart failure: new understandings of pathogenesis. Int J Biochem Cell Biol.

[6] Scott DL, Wolfe F, Huizinga TW (2010). Rheumatoid arthritis. Lancet.

[7] Wang R, Ward MM (2018). Epidemiology of axial spondyloarthritis: an update. Curr Opin Rheumatol.

[8] Lauper K, Gabay C (2017). Cardiovascular risk in patients with rheumatoid arthritis. Semin Immunopathol.

[9] Mackey RH, Kuller LH, Moreland LW (2018). Update on cardiovascular disease risk in patients with rheumatic diseases. Rheum Dis Clin North Am.

[10] Liao KP (2017). Cardiovascular disease in patients with rheumatoid arthritis. Trends Cardiovasc Med.

[11] El Maghraoui A (2011). Extra-articular manifestations of ankylosing spondylitis: prevalence, characteristics and therapeutic implications. Eur J Intern Med.

[12] Lam WC, Pennell DJ (2016). Imaging of the heart: historical perspective and recent advances. Postgrad Med J.

[13] Kumar A, Bagur R (2015). Cardiac magnetic resonance in clinical cardiology. World J Cardiol.

[14] Wintersperger BJ, Bamberg F, De Cecco CN (2015). Cardiovascular imaging: the past and the future, perspectives in computed tomography and magnetic resonance imaging. Invest Radiol.

[15] Houri Levi E, Watad A, Whitby A, Tiosano S, Comaneshter D, Cohen AD, Amital H (2016). Coexistence of ischemic heart disease and rheumatoid arthritis patients - a case control study. Autoimmun Rev.

[16] John H, Toms TE, Kitas GD (2011). Rheumatoid arthritis: is it a coronary heart disease equivalent?. Curr Opin Cardiol.

[17] Al-Mohaissen MA, Chan KL (2016). Echocardiography in the assessment of patients with rheumatologic diseases. Curr Cardiol Rep.

[18] Lazúrová I, Tomáš Ľ (2017). Cardiac impairment in rheumatoid arthritis and influence of anti-TNFα treatment. Clin Rev Allergy Immunol.

[19] Ntusi NAB, Piechnik SK, Francis JM, Ferreira VM, Matthews PM, Robson MD, Wordsworth PB, Neubauer S, Karamitsos TD (2015). Diffuse myocardial fibrosis and inflammation in rheumatoid arthritis: insights from CMR T1 mapping. JACC Cardiovasc Imaging.

[20] Kobayashi Y, Giles JT, Hirano M, Yokoe I, Nakajima Y, Bathon JM, Lima JA, Kobayashi H (2010). Assessment of myocardial abnormalities in rheumatoid arthritis using a comprehensive cardiac magnetic resonance approach: a pilot study. Arthritis Res Ther.

[21] Kobayashi H, Kobayashi Y, Yokoe I, Akashi Y, Takei M, Giles JT (2017). Magnetic resonance imaging-detected myocardial inflammation and fibrosis in rheumatoid arthritis: associations with disease characteristics and N-terminal pro-brain natriuretic peptide levels. Arthritis Care Res (Hoboken).

[22] Giles JT, Malayeri AA, Fernandes V, Post W, Blumenthal RS, Bluemke D, Vogel-Claussen J, Szklo M, Petri M, Gelber AC, Brumback L, Lima J, Bathon JM (2010). Left ventricular structure and function in patients with rheumatoid arthritis, as assessed by cardiac magnetic resonance imaging. Arthritis Rheum.

[23] Biesbroek PS, Heslinga SC, Konings TC, van der Horst-Bruinsma IE, Hofman MBM, van de Ven PM, Kamp O, van Halm VP, Peters MJL, Smulders YM, van Rossum AC, Nurmohamed MT, Nijveldt R (2017). Insights into cardiac involvement in ankylosing spondylitis from cardiovascular magnetic resonance. Heart.

[24] Biesbroek PS, Heslinga SC, van de Ven PM, Peters MJL, Amier RP, Konings TC, Maroules CD, Ayers C, Joshi PH, van der Horst-Bruinsma IE, van Halm VP, van Rossum AC, Nurmohamed MT, Nijveldt R (2018). Assessment of aortic stiffness in patients with ankylosing spondylitis using cardiovascular magnetic resonance. Clin Rheumatol.

[25] Castaneda S, Gonzalez-Juanatey C, Gonzalez-Gay MA (2018). Inflammatory arthritis and heart disease. Curr Pharm Des.

[26] Sivalingam Z, Larsen SB, Grove EL, Hvas AM, Kristensen SD, Magnusson NE (2017). Neutrophil gelatinase-associated lipocalin as a risk marker in cardiovascular disease. Clin Chem Lab Med.

[27] Katano M, Okamoto K, Arito M, Kawakami Y, Kurokawa MS, Suematsu N, Shimada S, Nakamura H, Xiang Y, Masuko K, Nishioka K, Yudoh K, Kato T (2009). Implication of granulocyte-macrophage colony-stimulating factor induced neutrophil gelatinase-associated lipocalin in pathogenesis of rheumatoid arthritis revealed by proteome analysis. Arthritis Res Ther.

[28] Bradham W, Ormseth MJ, Elumogo C, Palanisamy S, Liu CY, Lawson MA, Soslow JH, Kawel-Boehm N, Bluemke DA, Stein CM (2018). Absence of fibrosis and inflammation by cardiac magnetic resonance imaging in rheumatoid arthritis patients with low to moderate disease activity. J Rheumatol.

[29] Kobayashi Y, Kobayashi H, Hirano M, Giles JT (2014). Left ventricular regional dysfunction using cardiac magnetic resonance imaging in rheumatoid arthritis patients without cardiac symptoms: comparison between methotrexate and biologics treatment groups. J Rheumatol.

[30] Atzeni F, Turiel M, Caporali R, Cavagna L, Tomasoni L, Sitia S, Sarzi-Puttini P (2010). The effect of pharmacological therapy on the cardiovascular system of patients with systemic rheumatic diseases. Autoimmun Rev.

[31] Adler Y, Charron P, Imazio M, Badano L, Barón-Esquivias G, Bogaert J, Brucato A, Gueret P, Klingel K, Lionis C, Maisch B, Mayosi B, Pavie A, Ristić AD, Tenas MS, Seferovic P, Swedberg K, Tomkowski W (2015). 2015 ESC Guidelines for the diagnosis and management of pericardial diseases. Kardiol Pol.

[32] Azpiri-Lopez JR, Galarza-Delgado DA, Colunga-Pedraza IJ, Arvizu-Rivera RI, Cardenas-de la Garza JA, Vera-Pineda R, Davila-Jimenez JA, Martinez-Flores CM, Rodriguez-Romero AB, Guajardo-Jauregui N (2021) Echocardiographic evaluation of pulmonary hypertension, right ventricular function, and right ventricular-pulmonary arterial coupling in patients with rheumatoid arthritis. Clin Rheumatol. Epub ahead of print. 10.1007/s10067-020-05544-z 10.1007/s10067-020-05544-z 33443606

[33] Naseem M, Samir S, Ibrahim IK, Khedr L, Shahba AAE (2019). 2-D speckle-tracking assessment of left and right ventricular function in rheumatoid arthritis patients with and without disease activity. J Saudi Heart Assoc.

[34] Zungur M, Gul I, Kobak S (2018). Evaluation of right ventricular function by speckle-tracking echocardiography in patients with ankylosing spondylitis: a case-control study. Acta Cardiol Sin.

